# Experimental method for haplotype phasing across the entire length of chromosome 21 in trisomy 21 cells using a chromosome elimination technique

**DOI:** 10.1038/s10038-022-01049-6

**Published:** 2022-05-31

**Authors:** Sachiko Wakita, Mari Hara, Yasuji Kitabatake, Keiji Kawatani, Hiroki Kurahashi, Ryotaro Hashizume

**Affiliations:** 1 Department of Pathology and Matrix Biology, Mie University Graduate School of Medicine, Mie, Japan; 2 Department of Pediatrics, Graduate School of Medicine, Osaka University, Suita, Osaka Japan; 3 Division of Molecular Genetics, Institute for Comprehensive Medical Science, Fujita Health University, Toyoake, Japan; 4 Department of Genomic Medicine, Mie University Hospital, Mie, Japan; 5 Present Address: Department of Neuroscience, Mayo Clinic, Scottsdale, AZ USA

**Keywords:** Genotyping and haplotyping, Skin stem cells

## Abstract

Modern sequencing technologies produce a single consensus sequence without distinguishing between homologous chromosomes. Haplotype phasing solves this limitation by identifying alleles on the maternal and paternal chromosomes. This information is critical for understanding gene expression models in genetic disease research. Furthermore, the haplotype phasing of three homologous chromosomes in trisomy cells is more complicated than that in disomy cells. In this study, we attempted the accurate and complete haplotype phasing of chromosome 21 in trisomy 21 cells. To separate homologs, we established three corrected disomy cell lines (ΔPaternal chromosome, ΔMaternal chromosome 1, and ΔMaternal chromosome 2) from trisomy 21 induced pluripotent stem cells by eliminating one chromosome 21 utilizing the Cre-loxP system. These cells were then whole-genome sequenced by a next-generation sequencer. By simply comparing the base information of the whole-genome sequence data at the same position between each corrected disomy cell line, we determined the base on the eliminated chromosome and performed phasing. We phased 51,596 single nucleotide polymorphisms (SNPs) on chromosome 21, randomly selected seven SNPs spanning the entire length of the chromosome, and confirmed that there was no contradiction by direct sequencing.

## Introduction

The reconstruction of the two distinct copies of each chromosome (called haplotype phasing) has important implications in understanding human genetic variations [1–3]. Recent studies have shown that allele-specific expression is widespread in humans, and two groups showed that 1–5% of human genes are affected by cis-acting DNA sequence variants [4, 5]. In addition, several studies have highlighted the importance of haplotypes that might affect certain diseases, including tumorigenesis [6], bronchial asthma [7], sickle cell disease [8], and Fukuyama congenital muscular dystrophy [9]. Moreover, according to Lawson DJ et al., the analysis of human genome diversity data by similar haplotype patterns can capture information about human population structures that reflect continental-, regional-, local-, and family-scale differences, and can directly reveal important information about ancestral relationships among individuals [10]. However, conventional methods are only able to interrogate variants and do not provide phased information, as it is not known whether the two variants are on the same chromosome (cis) or different chromosomes (trans).

Next-generation sequencing (NGS) has been used for haplotype phasing. Advances in massive parallel sequencing technologies have considerably lowered the cost of human whole-genome sequencing [11]. However, the short read lengths created by technologies such as Illumina HiSeq (100–250 bases) make it impracticable to link distant variants into haplotypes [12]. To overcome this limitation, methods to preserve information from long DNA fragments (tens to hundreds of kilobases) in short sequence reads have been developed [13]. Recently, 10X Genomics has described a novel approach that generates long-linked readings that can be combined into long haplotypes [14]. Third-generation sequencing technologies, such as Pacific Bioscience (PacBio), generate long sequence readings (2–20 kb in length) that can directly allow genome-wide haplotype phasing. Unfortunately, an increased length results in decreased data accuracy [15]. In the case of haplotype phasing in human samples, in addition to read accuracy, high coverage is required to reduce possible errors due to the few reads that convey conflicting information [16].

To overcome the above challenges, current approaches for haplotype phasing include using emulsion PCR to condense polymorphic sites from a single template [17], genotyping from diluted aliquots of DNA fragments [18], allele-specific imaging of long-range PCR products [19], genotyping from sperm [20], and isolation of single chromosomes by interspecific cell fusion [21]. Most of these methods have been designed for only a few markers [22]. Exceptions are the execution of single nucleotide polymorphism (SNP) table profiling after chromosome microdissection [23], single-stranded sequencing using microfluidic reactors (SISSOR) [24], and diploid assembly (DipAsm) [25]. In the new microfluidics-based technology SISSOR, the DNA of a single cell is isolated and denatured, which also breaks up the DNA into megabase-sized fragments. DipAsm uses long reads and long-range confirmation data from a single individual to generate a chromosome-scale phased assembly. However, assembly accuracy may be reduced [25], and this method is still relatively complex and expensive [26]. Furthermore, generating chromosome-level haplotype phasing in trisomy cells remains a challenge.

Multiple sequencing technologies and protocols can generate sequence reads with haplotype information but require computational tools to assemble the reads into long haplotypes. Several combinatorial algorithms have been developed for haplotype phasing [1]. More recently, several algorithms have been designed to enable haplotype phasing from long reads [27]. However, these methods remain speculative [2] and are designed only for disomy and not trisomy.

**Fig. 1:**
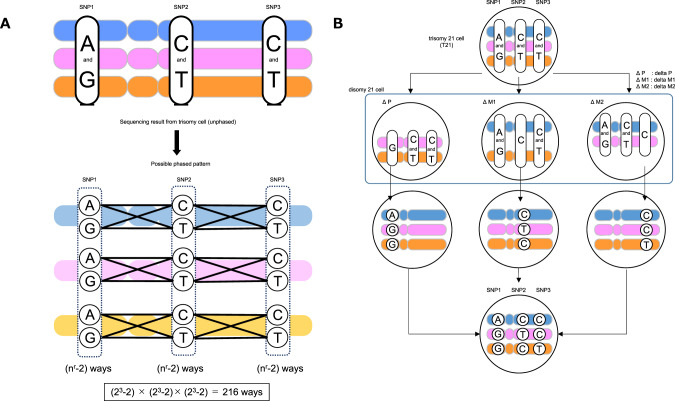
Overview of haplotype phasing. **A** A scheme showing three SNPs and their combinations in trisomy cells. In this example, there are theoretically 216 combinations of the three single nucleotide polymorphisms (SNPs) on the three chromosomes. Any given allele cannot be distinguished from others, which makes haplotype phasing difficult. **B** Diagram explaining the haplotype phasing method in trisomy cells in the study. The three corrected disomy cells are used, in each of which one chromosome is deleted from trisomy cells. By comparing the base information of the WGS data at the same position between each corrected disomy cell line, the base on the erased chromosome can be determined if the corrected disomy is homozygous at that position

Haplotype phasing in trisomy cells is more complicated than that in disomy cells. The three chromosome copies in trisomy cells have four possible genotypes for biallelic markers: AAA, AAB, ABB, and BBB. The two heterozygous groups (i.e., AAB and ABB) are indistinguishable, which makes the phasing procedure difficult (Fig. 1A). If it is possible to phase three chromosomes and their epigenetic differences, this information could help us identify expression patterns that cause an array of phenotypes based on aneuploidy disorders.

Here, we propose a method that completes haplotype phasing across the entire chromosome for the three chromosome 21’s of a trisomy cell. Our method compares the base information from whole-genome sequence (WGS) data obtained from the original trisomy 21 cells and three corrected disomy cells. Although some techniques have been reported by other groups for haplotype phasing of trisomy cells [28, 29], our method is speculation-free, reliable, and reproducible.

## Materials and methods

### Ethics statement

This study was performed according to the Declaration of Helsinki and was approved by the Mie University Medical Research Ethics Committee (approval number: 1578). Selection of an individual with Down syndrome who has karyotype (47, XY, + 21) and the procedures for dermal sampling, isolation, and expansion of dermal fibroblasts were performed following an approved protocol. The purpose and content of this study were explained to the parents of the cell donors orally and in writing, and informed consent was obtained. All protocols used for animal experiments in this study were approved by the Animal Experimentation Committee of Mie University (approval number: 29–26). The study was conducted in compliance with the ARRIVE guidelines.

### Reprogramming of skin fibroblasts and cell culture

Approximately 1 mm^3^ of dermal tissue was harvested from a boy with Down syndrome at the time of orthopedic surgery following informed consent. Dermal tissue was sandwiched between two coverslips in a 35 mm dish and cultured in Dulbecco’s Modified Eagle’s Medium (Gibco, Thermo Fisher Scientific, Massachusetts, USA) with 10% fetal bovine serum (PAA Laboratories GmbH, Upper Austria, Austria) at 37 °C and 5% CO_2_ until fibroblasts migrated out of the tissue. The fibroblasts were passaged weekly. At passage 5, 6 × 10^5^ cells were electroporated with episomal plasmid vectors encoding hOCT4, hSOX2, hKLF4, hL-MYC, hLIN-28, short hairpin RNA for TP53 (shp53), and EBNA-1 (Addgene plasmids #27077, #27078, #27080, and #37624) using the Neon transfection system (Invitrogen, Massachusetts, USA) [30, 31]. On day 7, the cells were passaged, and 1 × 10^5^ cells were plated onto a 100 mm tissue culture dish coated with 1.5 × 10^6^ SNL76/7 feeder cells (DS Pharma Biomedical, Osaka, Japan) treated with mitomycin C (Wako, Osaka, Japan). The next day, the culture medium was replaced with Primate ES Cell Medium (Reprocell, Kanagawa, Japan). On day 25–31 post-transduction, embryonic stem cell-like colonies positively stained by rBC2LCN antibody (#180–02991, Wako, Osaka, Japan) were picked up and passaged onto new wells of a 24-well-plate in feeder-free conditions [32] with StemFit AK03 medium (Ajinomoto, Tokyo, Japan) containing 10 µM Y-27632 (Fujifilm Wako Pure Chemical Corporation, Osaka, Japan) and 0.4 µg/mL iMatrix-511 (Nippi, Tokyo, Japan). StemFit AK03 without Y-27632 and iMatrix-511 was used for the standard induced pluripotent stem cells (iPSC) culture. Of the 24 colonies, we selected one colony (original trisomy: T21) based on cell morphology, lower differentiation tendency, and staining properties with an anti-Tra-1–60 antibody (#09–0068, Stemgent, Cambridge, MA). The cells were cultured according to the protocols for human iPSC culture under feeder-free conditions provided by the Center for iPS Cell Research and Application (Kyoto University) [30].

### Cell culture

The iPSCs generated from primary human dermal fibroblasts were maintained in 0.4 µg/mL iMatrix-511, using StemFit AK02N medium supplemented with 10 µM Y-27632. To subculture human iPSCs, cells were treated with TrypLE (Life Technologies, NY, USA) at 37 °C for 4 min and scraped off from the wells. After centrifugation at 200 × *g* for 5 min, the cells were seeded onto a new well with 0.4 µg/mL iMatrix-511 at a density of 5 × 10^4^ cells/cm^2^. The cells were subcultured every 2–6 days.

### Teratoma formation

The cells were washed with PBS, scraped, and collected. Approximately 2 × 10^6^ cells in 100 µL Matrigel (BD Biosciences, New Jersey, USA) were intramuscularly injected into the thigh of a 9-week-old immunodeficient NOD/SCID mouse under 2% isoflurane (Fujifilm Wako Pure Chemical Corporation, Osaka, Japan) and 100% oxygen anesthesia. Ten weeks after injection, the formed mass was dissected and fixed in 4% (w/v) paraformaldehyde (Merck Corporation, Tokyo, Japan). The tissue was embedded in paraffin, sectioned, and analyzed histologically by hematoxylin and eosin (H&E) staining [33]. Images were captured using an optical microscope (Keyence VHX-800; Osaka, Japan).

### Short tandem repeat analysis

To identify the origin of chromosome 21 in trisomy dermal fibroblasts, short tandem repeat (STR) analysis was adapted. Genomic DNA was extracted from the dermal fibroblasts with trisomy 21. Next, DNA samples were obtained by scraping the oral mucosa from the parents. The parental DNA samples were only used for STR analysis to determine the parental origin of chromosome 21 in the trisomy cell line. Genomic DNA was extracted using the QuickExtract DNA Extraction Solution (Lucigen, Wisconsin, USA) according to the manufacturer’s protocol. Multiplex PCR was performed using PrimeSTAR Max DNA polymerase (Takara Bio Inc., Shiga, Japan) with a primer set that amplified the STR loci Penta-D, D21S11, and D21S1411 [34, 35] on chromosome 21. For fragment analysis, we used capillary electrophoresis on the ABI 3130 Genetic Analyzer (Applied Biosystems, Massachusetts, USA). One microliter of PCR product was added to 8.5 µL Hi-Di Formamide (Invitrogen, Massachusetts, USA) and 0.5 µL of Internal Lane Standard 600 size standard (Promega, Wisconsin, USA). After data collection, samples were analyzed using GeneMapper v.4.0 software (Applied Biosystems, Massachusetts, USA). Supplementary Table 1 shows the primer sequences, PCR program, and product sizes used in this study.

### Fabrication of disomy 21 iPSCs

Fabrication of disomy 21 iPSCs was accomplished by co-electroporating the targeting vector and sgRNA/Cas9 expression vector into trisomy 21 iPSCs, followed by positive/negative drug selection. An *HSF2BP* intron 3-specific CRISPR/Cas9 expression vector was constructed in eSpCas9(1.1)-pX330 containing expression cassettes for *Streptococcus pyogenes* Cas9 nuclease (Addgene #71814). The Cas9 target site on *HSF2BP* intron 3 (5′-GAGATTGCCTATCGTAGAGTGGGNGG-3′) is located 10 kb downstream of the Penta-D STR locus. In addition, we constructed a “chromosome elimination cassette” (2797 bp) containing a CAG promoter-driven puromycin-delta thymidine kinase (puroΔTK) flanked by inverted loxP sites to bear positive/negative drug selection markers [36, 37] (using puroΔTK fragment from Addgene #84036). The targeting vector (6451 bp DNA plasmid) designed with homology arms against *HSF2BP* intron 3 (973 and 982 bp in length) (Supplementary Fig. S1) was constructed using the NEBuilder HiFi DNA Assembly system (New England Biolabs Japan, Tokyo, Japan). On the day of transfection (day 0), 5 × 10^5^ cells were dissociated with TrypLE, mixed with CRISPR/Cas9 expression vector (2 µg) and the targeting vector (6 µg), and then electroporated using the Neon Transfection System (Invitrogen, Massachusetts, USA). On day 4, drug selection with puromycin (0.5 µg/mL) was initiated, and the resulting colonies were selected on days 9–10. Junction PCR was performed to test for the elimination cassette integrated at the correct locus. The parental origin of the knocked-in allele was analyzed using STR analysis. The clones were then subjected to Cre recombinase-mediated chromosome elimination (Addgene #13775), followed by negative FIAU selection. After single cells were cloned by limiting dilution, we isolated three types of disomy 21 iPSCs: ΔPaternal chromosome (ΔP), ΔMaternal chromosome 1 (ΔM1), and ΔMaternal chromosome 2 (ΔM2). The isolated colonies were expanded for fluorescence in situ hybridization (FISH), STR, G-band karyotype analysis, NGS, Sanger sequencing, and multiplex ligation-dependent probe amplification (MLPA) analysis.

### FISH

We used chromosome 21-specific probe 1 (BAC clone RP11–15E10) and probe 2 (BAC clone RP11–777J19), which were hybridized to 21q21.1 and 21q22.13, respectively. The cells were attached to a glass slide treated with pre-warmed denaturation buffer at 72 °C for 2 min and placed in 70% (w/v) ethanol at 4 °C, and 90% (w/v) and 100% (w/v) ethanol at room temperature (20 to 25 °C) for 5 min. Before hybridization, probes were denatured at 80 °C for 10 min, placed at 37 °C for 30 min, and then applied to the slides, which were then incubated at 37 °C for 16–20 h for hybridization. After washing, the slides were visualized under a microscope using an appropriate fluorescent filter.

### G-banding karyotyping

The cells were treated with 0.02 µg/mL colcemid (Gibco, NY, USA) for 2 h to enrich metaphase cells, exposed to buffered hypotonic solution (Genial Helix, Flintshire, UK) for 15 min at 37 °C, and fixed thrice with 3:1 methanol: acetic acid for 5 min each at room temperature. The fixed cells were sent to Chromocenter, Inc. (Tottori, Japan) for high-resolution G-banded karyotyping.

### Whole-genome sequencing (WGS)

Genomic DNA was extracted from cell pellets using the QIAprep Spin Miniprep Kit (QIAGEN, Venlo, The Netherlands) and sent to Hokkaido System Science Co. (Hokkaido, Japan). Paired-end 150 base-pair read lengths were sequenced on an Illumina HiSeq X next-generation sequencer, achieving 30-fold coverage on average per sample. The Burrows–Wheeler Aligner (BWA version. 0.7.8-r455) was used to map the paired-end clean reads to the human reference genome (b37, ftp://gsapubftp-anonymous@ftp.broadinstitute.org/bundle/b37/human_g1k_v37_decoy.fasta.gz). The BAM files were viewed using Integrative Genomics Viewer [38], and the SNPs on chromosome 21 were identified using Samtools (version. 1.0) [39]/BCFtools (version. 1.2) [40]. SnpEff [41] was used for annotating genetic variants and creating mutation table data.

### Comparison of the combination of SNP data between each corrected disomy cell

Four cellular strains with different combinations of chromosome 21 (T21, ΔP, ΔM1, and ΔM2) were subjected to whole-genome sequencing. Based on the aligned data (BAM format) for four samples obtained from WGS, multi-sample variant calling focusing on chromosome 21 was performed. Among the four samples, SNV locations covered by at least 10 or more reads per sample were extracted. In addition, from the variant data of chromosome 21, variants detected as insertions and deletions (indel) were excluded. In an effort to resolve their haplotypes across chromosome 21, we first calculated a ratio of bases (base allele frequency) at each heterogeneous SNV locus for each cell line, based on the number of the aligned sequence reads. From the variant data, only the variant information in which some bases were detected at a ratio of 0.06 or more in T21 cells with three chromosomes (P, M1, and M2) was extracted to filter base call errors. Theoretically, at any given position, bases specific to the allele disappear only in the cell line in which the allele has been deleted. Theoretically, at any position, bases specific to the allele disappear only in the cell line that the allele has been deleted. Therefore, we selected allele-specific bases for each chromosome 21 using conditions summarized in Supplementary Table 2. By simply comparing the base sequence at the same position, we determined the DNA sequence on the erased chromosome and performed phasing. For example, the base at SNP1 of each T21, ΔM1, and ΔM2 cell has two types of bases: adenine (A) and guanine (G), while ΔP cells have only G, suggesting that SNP1 of P allele is A and those of M1 and M2 alleles are G (Fig. 1B).

### Sanger validation and segregation analysis

The primers were designed to include the identified SNP regions using Primer3 online software [42]. PCR was performed using PrimeSTAR Max, according to the manufacturer’s protocol. The PCR products (product size: 306–1 509 bp) were analyzed by Sanger sequencing using an ABI 3130XL DNA Analyzer. The sequencing data were used to compare trisomy cells with those of the corrected disomy cells.

### MLPA analysis

Genomic DNA was analyzed using the SALSA MLPA probe-mix P095/Aneuploidy kit (MRC-Holland, Amsterdam, Netherlands), according to the manufacturer’s instructions. The product fragments were separated by capillary electrophoresis on an ABI 3130XL DNA analyzer. MLPA data were analyzed using Coffalyser.Net v.140721.1958 (MRC-Holland, Amsterdam, Netherlands). Following the manufacturer’s instructions, a trisomy was indicated if at least four of the eight relative probe ratios were ≥1.30 when compared with the control for a certain chromosome [43].

### Depositing resources

We deposited the trisomy 21 iPS cell line [47, XY, + 21] (identification number HPS4270) and three types of corrected disomy 21 iPS cell line [46,XY] (identification numbers HPS4271, HPS4272, and HPS4273) at the RIKEN BioResource Research Center (Ibaraki, Japan).

## Results

### Teratoma formation assay

We transplanted a NOD/SCID mouse with iPSCs and prepared a tumor isolated from the mouse thigh for histological analysis (Supplementary Fig. S2a). H&E staining showed three germ layer differentiation (endoderm, mesoderm, and ectoderm), and no formation of malignant neoplasms (Supplementary Fig. S2b–i). The endodermal epithelium (Supplementary Fig. S2c), mesodermal derivatives (Supplementary Fig. S2d), mesodermal cartilage (Supplementary Fig. S2e), cartilage and bone trabecula (Supplementary Fig. S2f), smooth muscle tissue (Supplementary Fig. S2g), ectodermal melanocytes (Supplementary Fig. S2h), neuroepithelia (Supplementary Fig. S2i), and neural tube-like structures (Supplementary Fig. S2j) are shown.

### Induction of disomy 21 from iPS cells with trisomy 21

At the Penta-D STR locus, three alleles of dermal fibroblasts, which were trisomy 21, consisted of one paternal origin allele (P: repeat #9) and two maternal origin alleles (M1: repeat #10, M2: repeat #13) of chromosome 21 (Supplementary Fig. S3). Results compatible with the Penta-D locus were achieved for other loci (D21S11 and D21S1411 (data not shown)).

**Fig. 2:**
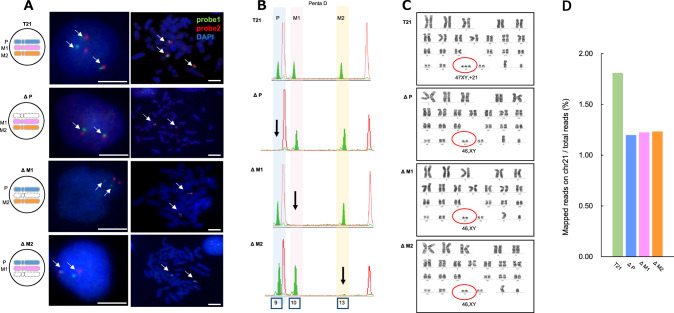
Characterization of trisomy 21 cells and disomy cells. **A** Representative fluorescence in situ hybridization (FISH) images at metaphase and interphase. Arrows indicate the locus of probe1 (21q21.1: green) and probe2 (21q22.13: red) on chromosome 21. DNA was counterstained by DAPI. Scale bars: 10 µm. **B** The short tandem repeat (STR) analysis at the Penta-D locus on chromosome 21 defined the eliminated allele. The three corrected disomy cell lines are referred to as Δ followed by the origin of eliminated homologous chromosome (i.e., ΔP, ΔM1, and ΔM2). **C** Trisomy 21 induced pluripotent stem cells (iPSCs) generated from human skin fibroblasts had a trisomy 21 karyotype (47, XY, + 21). The corrected disomy cells (ΔP, ΔM1, and ΔM2) had a euploid (46, XY) karyotype. **D** The *y* axis is the count number of mapped reads on chromosome 21 from NGS data normalized by total reads. These data indicated that one copy of chromosome 21 had been successfully eliminated from the original trisomy cells, and the karyotype converted to disomy. T21; original trisomy 21 cells

Three types of disomy 21 cell lines, corrected from the original trisomy 21, were established by eliminating each chromosome 21 using the chromosome elimination cassette with the Cre-loxP system (Supplementary Fig. S1). The elimination efficiency of one chromosome 21 in the trisomy iPSCs by the Cre-loxP system was 3.48% (disomy colonies evaluated by STR analysis/total picked-up colonies: 12/345) for ΔP, 3.57% (6/168) for ΔM1, and 3.13% (3/96) for ΔM2. Figure 2 shows a summary of the genetic profiles of the corrected disomy 21 cells.

Analysis of chromosome 21 in the interphase nuclei and metaphase spread was performed using FISH. In trisomy cells, three copies of chromosome 21 (three red and three green signals; six signals in total) were detected (T21 cell in Fig. 2A). In the corrected disomy cells, two copies of chromosome 21 (two red signals and two green signals; four signals in total) were detected (ΔP, ΔM1, and ΔM2 cells in Fig. 2A).

Based on STR analysis, three strains (ΔP, ΔM1, and ΔM2) with different origin patterns of chromosome 21 were selected. Figure 2B shows three alleles in the trisomy and the deletion of one allele in each corrected disomy 21 cell. The T21 cell had three alleles: P, M1, and M2; the ΔP cell had two alleles, M1 and M2; the ΔM1 cell had two alleles, P and M2; and the ΔM2 cell had two alleles, P and M1.

Genomic integrity was assessed using G-band analysis. The trisomy 21 cell line used in this study retained full trisomy 21 (i.e., 47, XY, + 21) (Fig. 2C). In contrast, the three types of corrected disomy 21 cell lines (ΔP, ΔM1, and ΔM2) retained full disomy 21 (i.e., 46, XY) without any acquired cytogenetic aberrations (Fig. 2C). We confirmed that there were no structural abnormalities at the 5 Mb level using G-banding.

The total genomic DNA extracted from T21 cells and three corrected disomy 21 cell lines (ΔP, ΔM1, and ΔM2) was analyzed by whole-genome sequencing. Mapped reads on chromosome 21 per total reads were reduced by approximately one-third in all corrected disomy cells compared with those in the T21 cells (mapped reads on chromosome 21 per total reads were 12 834 391/709 100 730 (1.81%) for T21, whereas this number was 1.20% for ΔP, 1.16% for ΔM1, and 1.23% for ΔM2) (Fig. 2D).

MLPA analysis results showed that T21 cells had full trisomy 21. We determined the average relative probe signal for each chromosome 21-specific probe in the corrected disomy 21 cell lines (ΔP, ΔM1, and ΔM2). They showed an average relative probe signal of about 1.0 (ranges from 0.96–1.04). The three corrected disomy cells were negative for aneuploidies on chromosomes X, Y, 13, 18, and 21. All MLPA results were compatible with the results of FISH, STR analysis, and G-band analysis (Supplementary Fig. S4).

### Haplotype phasing

We successfully phased 51,596 SNPs on chromosome 21 in trisomy 21 cells (Supplementary Table 3). Seven SNPs spanning the entire chromosome were extracted and validated by direct sequencing. The seven SNP loci in which only one of three corrected disomy cells was homogeneous were randomly selected across the entire chromosome 21.

**Fig. 3:**
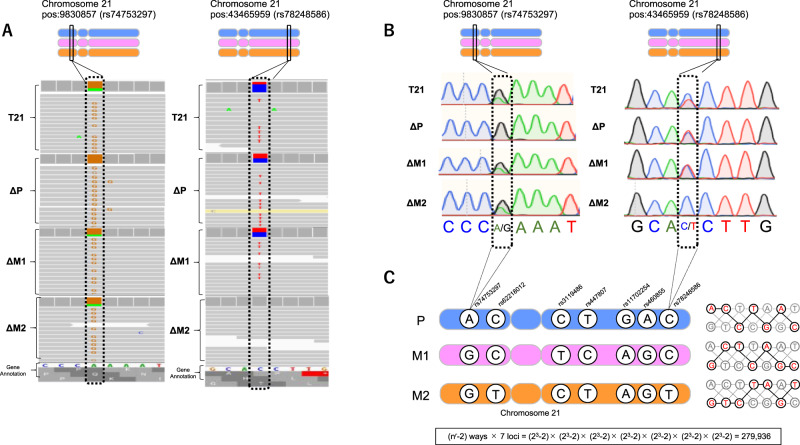
Part of the sequence and haplotype phasing results. **A** The whole-genome sequence (WGS) data were shown by Integrative genomics viewer (IGV) software as a short stretch of DNA on the same positions in chromosome 21 mapped onto GRCh37. Each SNP has two different bases; the rs74753297 has bases G and A, and the rs78248586 has bases C and T in the trisomy cells. By comparing the base information of the WGS data at the same position between each corrected disomy cell line, the base on the erased chromosome was determined. Note that T21, ΔM1, and ΔM2 cells harbor both A and G, while ΔP cells have only G at the rs74753297 position, and T21, ΔP, and ΔM1 cells harbor both C and T, while ΔM2 has only C at the position of rs78248586. **B** The results of Sanger sequencing supported those from WGS at the positions rs74753297 and rs78248586. **C** Seven phased SNPs that extend across the centromere of chromosome 21 in trisomy 21 cells. T21: Original trisomy 21 cells

From the comparison of the mapped BAM files, the nucleotide at pos: 9830857 in GRCh37.v16 (rs74753297) of each T21, ΔM1, and ΔM2 cell had two types of bases: adenine (A) and guanine (G) on the positive strand, whereas ΔP cells had only G, indicating that rs74753297 of the P allele was A and those of the M1 and M2 alleles were G (Fig. 3A, left). Similarly, each pos:43465959 in GRCh37.v16 (rs78248586) of T21, ΔP, and ΔM1 cells had two alleles, cytosine (C) and thymine (T); whereas the rs78248586 of ΔM2 had only C, indicating that rs78248586 of the M2 allele was T and those of the P and M1 alleles were C (Fig. 3A, right).

Using Sanger sequencing, we found that the base of rs74753297 was A/G on the positive strand in T21, ΔM1, and ΔM2 cells. In contrast, the base of rs74753297 was only G in ΔP cells. Therefore, the base of rs74753297 in the P allele was identified as A, and the same SNPs in the M1 and M2 alleles were identified as G (Fig. 3B, left). Similarly, the base of rs78248586 was C/T in T21, ΔP, and ΔM1 cells. In contrast, the base of rs78248586 was only C in ΔM2 cells. Accordingly, the base of rs78248586 in the M2 allele was identified as T and the same SNP in the P and M1 alleles was identified as C (Fig. 3B, right).

The same analysis was performed for the remaining five loci. By comparing the results of the NGS method with Sanger sequencing, a perfect agreement was observed between the two methods (Fig. 3A, B).

Given the information above, the particular combinations of seven SNPs (in this order), rs74753297 and rs62218012 in the short arm sandwiches the centromere, and for the long arm (rs3119486, rs447807, rs11702254, rs460855, and rs78248586), it can be concluded that A-C-C-T-G-A-C is the P allele, G-C-T-C-A-G-C is the M1 allele, and G-T-C-T-A-G-T is the M2 allele (Fig. 3C).

A point histogram with 10,000 base bins for the number of detected heterozygous SNPs on chromosome 21 is shown in Supplementary Fig. S5. Comparison of the three chromosomes 21 revealed that M1 and M2 alleles are genetically close. However, as regions with homozygous SNPs pattern in any induced disomy, especially in the ΔP cell line, were not observed, it was inferred that no recombination of chromosome 21 occurred during the first meiosis of the oocytes of the cells used in this study.

## Discussion

Kleinjan & Coutinho made several statements about the importance of haplotype phasing, as follows [44]. First, the disruption of the cis-trans regulatory systems of a gene can adversely affect gene expression and lead to disease. Second, when a gene has multiple harmful alleles in the same person or cancer, determining whether the allele resides on the same chromosomal copy (cis-phenotype) or on the opposite copy (trans-phenotype, the potential to inactivate both copies) is significant for genetic analysis. Finally, the effective use of allele-specific expression analysis requires information on chromosomal location to evaluate the increase or decrease in expression. Therefore, methods for haplotype phasing will not only facilitate these tasks but will also be critical for both research and clinical applications [45]. However, it is currently difficult to determine precise haplotype phasing across the entire chromosome length, and it is even more difficult in trisomy cells. In this study, we present a novel method to solve the haplotype phasing problem in trisomy cells, a method that does not rely on inference. Moreover, a study has reported that in trisomy 21, heterozygotes for SNPs in chromosome 21 may interact with other genotypes, and a higher incidence of certain haplotypes were detected in persons with Down syndrome compared with those from euploid control individuals [46].

Down syndrome is caused by a numerical excess of chromosome 21. There is currently no safe and efficient method to eliminate the excess chromosomes from cells. The chromosome shredding using CRISPR/Cas9 platform (i.e., induction of multiple double-strand breaks in the targeted chromosome) has been reported as a method to eliminate these chromosomes in vitro [47, 48]. However, the method does not distinguish between homologous chromosomes; therefore, Zuo et al. argued that targeting only one of the homologous chromosomes based on single nucleotide polymorphisms would avoid undesirable results [43]. We also believe that it is preferable to target one of the three chromosomes to avoid potential genomic imprinting diseases. For attempting to eliminate the excess chromosome in human trisomy 21 iPSC by shredding only the target homologous chromosome using the CRISPR/Cas system, haplotype phasing is critically essential to identify each chromosome in advance.

Separating a single chromosome is not an easy task using current sequencing technologies. Therefore, haplotype phasing at the chromosome level remains challenging [49]. Statistical phasing or phasing based on alternative methods, such as linked reads or long reads, have been attempted [50]; however, this inevitably results in imprecise phasing. Furthermore, it is much more difficult in trisomy cells because the haplotypes of the three chromosomes are confounded [29].

To address this issue, we have developed a novel haplotype phasing method for trisomy cells. To separate the homologs, we successfully established three corrected disomy 21 iPS cell lines by introducing the Cre-loxP system [36, 51] into chromosome 21 at intron 3 of the *HSF2BP* gene in the original trisomy 21 iPSCs using CRISPR-Cas9 technology. These three types of cells have different combinations of 21 chromosomes. Karyotypes of the corrected disomy cells were confirmed by chromosome spreading G-banding, STR, MLPA, and FISH analyses. WGS was performed on the Illumina HiSeq X platform using genomic DNA isolated from four cellular strains with different combinations of chromosome 21 (T21, ΔP, ΔM1, and ΔM2). Quality trimmed Illumina reads were mapped to the human reference genome GRCh37 using BWA, and SNPs on chromosome 21 were identified using Samtools and BCFtools. By comparing the base information of the WGS data at the same position between each corrected disomy cell line, we determined the base on the erased chromosome and thereby performed phasing. As there are chromosome-specific SNPs in every region of the distribution map of the allele-specific SNP locus, it was inferred that the trisomy cells used in this study consisted of three chromosomes in which recombination did not occur during the first meiosis.

Our method not only enables phase alignment of the entire length of chromosome 21 in trisomy 21 cells without resorting to inference but also provides higher accuracy and longer haplotypes than other methods. In addition, since the three already established corrected disomy cell lines are available, the results of this method can be reproduced and validated by using other methods. Furthermore, although our method was only used on trisomy 21 cells, it may also be applied to cells from other aneuploid diseases, including Patau syndrome (trisomy 13), Edwards syndrome (trisomy 18), and Klinefelter syndrome (XXY).

Our method can phase haplotypes precisely over the entire length of chromosome 21 at the single nucleotide level. However, our method requires living cells, arduous processes including the cloning procedure to obtain corrected disomy cell lines, and specialized equipment for genome-editing technology. In other words, these requirements are costly and time-consuming. In addition, the method used in this study is based on the BWA mapper; therefore, mapping failures may have a significant effect on haplotype phasing failures.

In summary, we phased 51,596 SNPs, randomly selected seven SNP loci extending across chromosome 21 spanning the p-arm through the q-arm, and confirmed that there was no contradiction by direct Sanger sequencing. In trisomy cells, even if there were only seven SNP loci examined, there are theoretically 279,936 combinations of bases on the three chromosomes, yet our method was able to identify one of these combinations. We expect that our method of haplotype phasing and trisomy-derived disomy iPS cell lines will provide a useful resource for studying human diseases associated with aneuploidy.

## Supplementary information

Supplementary Fig.S1

Supplementary Fig.S2

Supplementary Fig.S3

Supplementary Fig.S4

Supplementary Fig.S5

Supplementary Table 1

Supplementary Table 2

Supplementary Table 3
