# Supplementary material for: Experimental method for haplotype phasing across the entire length of chromosome 21 in trisomy 21 cells using a chromosome elimination technique
Source: J Hum Genet. 2022 May 31;67(10):565–72. doi: 10.1038/s10038-022-01049-6 (PMC9510051; doi:10.1038/s10038-022-01049-6)
Supplement: Supplementary file 1 — Supplementary Fig.S1 [file 10038_2022_1049_MOESM1_ESM.pptx]

## Slide 1
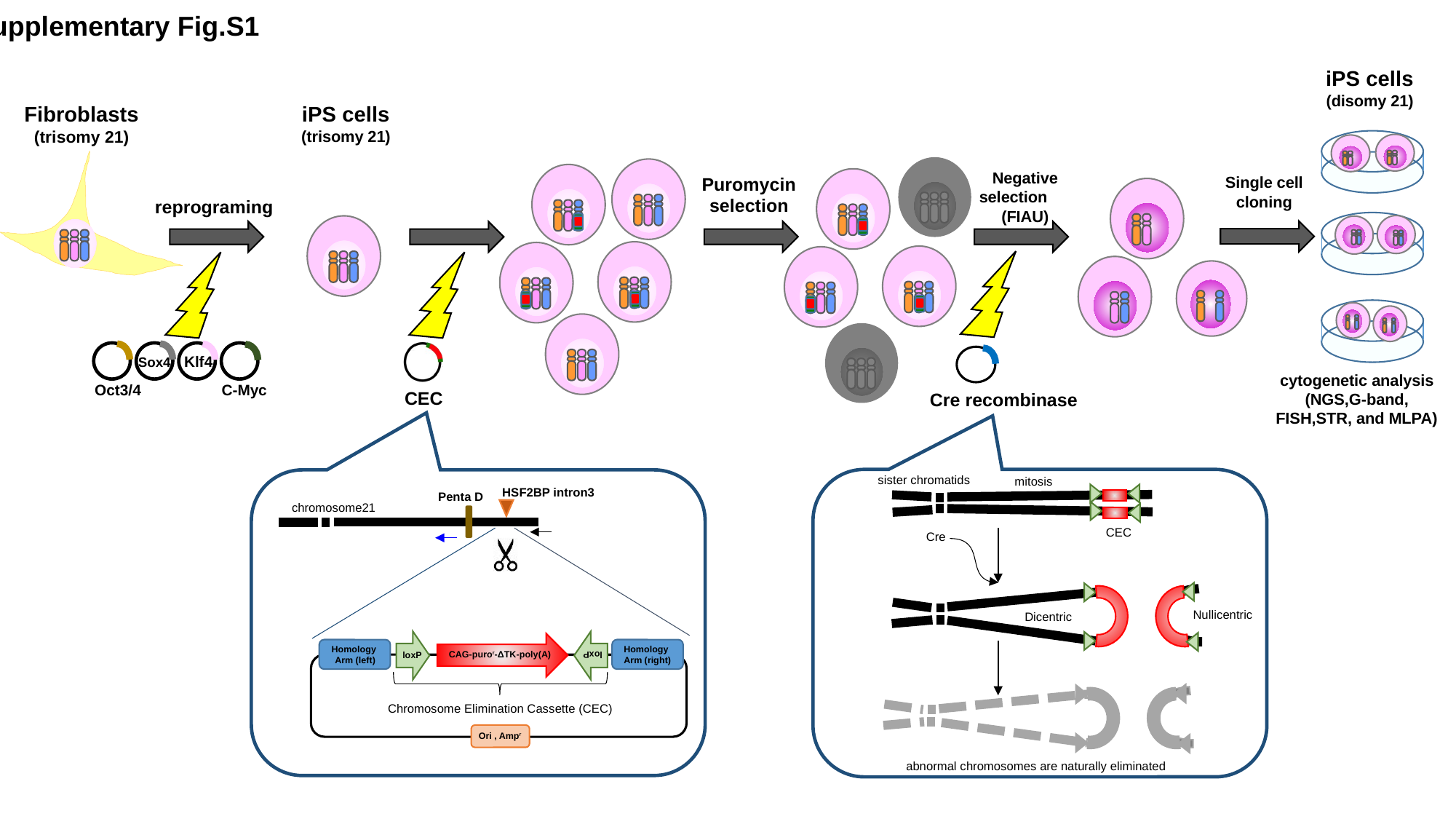

Supplementary Fig.S1
iPS cells
(disomy 21)
Fibroblasts
(trisomy 21)
iPS cells
(trisomy 21)
Negative selection　(FIAU)
Puromycin
selection
Single cell cloning
reprograming
Klf4
Sox4
cytogenetic analysis
(NGS,G-band,
FISH,STR, and MLPA)
Oct3/4
C-Myc
CEC
Cre recombinase
sister chromatids
mitosis
HSF2BP intron3
Penta D
chromosome21
CEC
Cre
Nullicentric
Dicentric
Homology
Arm (left)
Homology
Arm (right)
CAG-puror-ΔTK-poly(A)
loxP
loxP
Chromosome Elimination Cassette (CEC)
Ori , Ampr
 abnormal chromosomes are naturally eliminated
