# Supplementary figures and images for: Experimental method for haplotype phasing across the entire length of chromosome 21 in trisomy 21 cells using a chromosome elimination technique
Source: J Hum Genet. 2022 May 31;67(10):565–72. doi: 10.1038/s10038-022-01049-6 (PMC9510051; doi:10.1038/s10038-022-01049-6)

## Slide 1
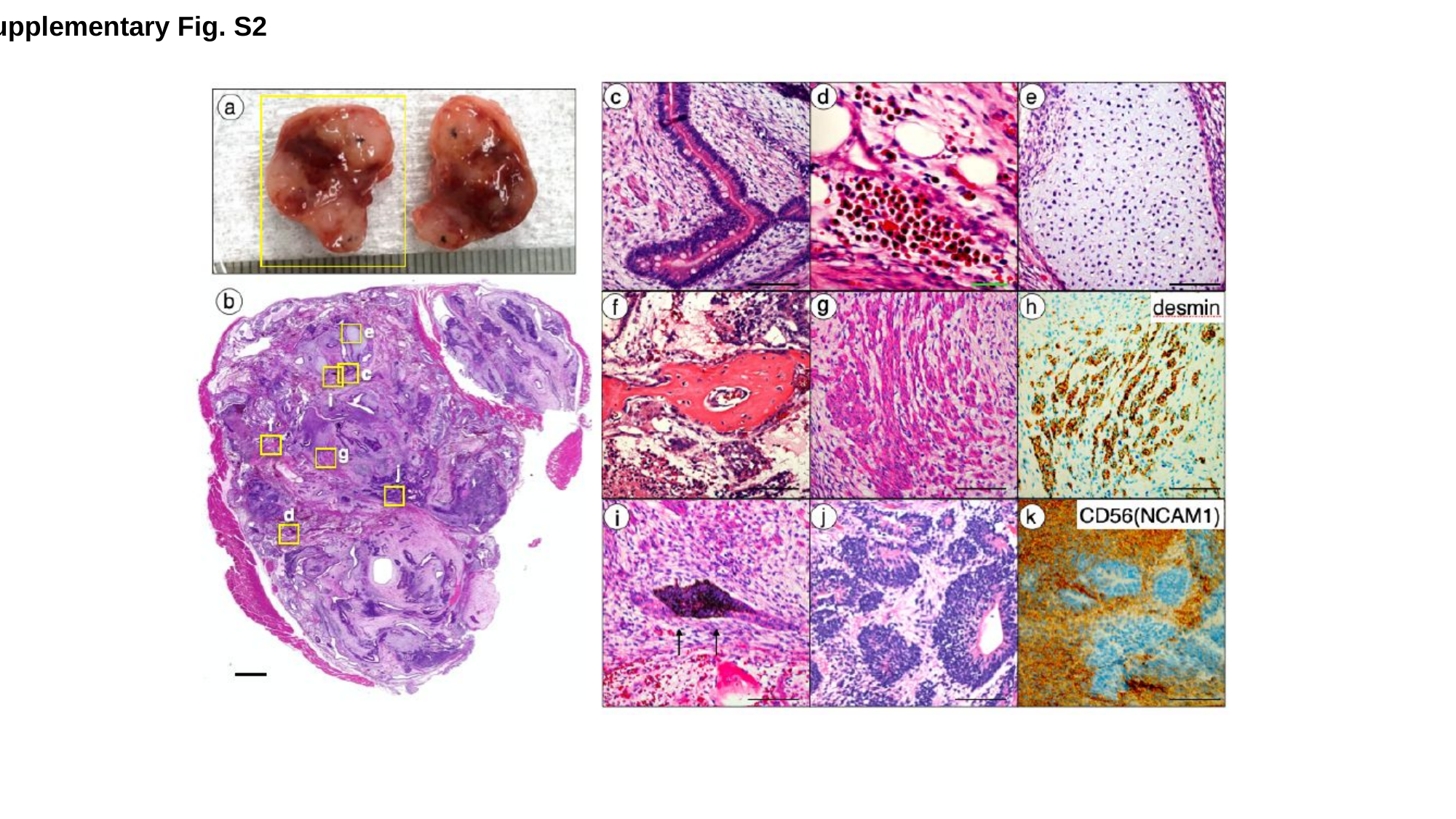

Supplementary Fig. S2

Supplement: Supplementary file 2 — Supplementary Fig.S2 [file 10038_2022_1049_MOESM2_ESM.pptx]

## Slide 1
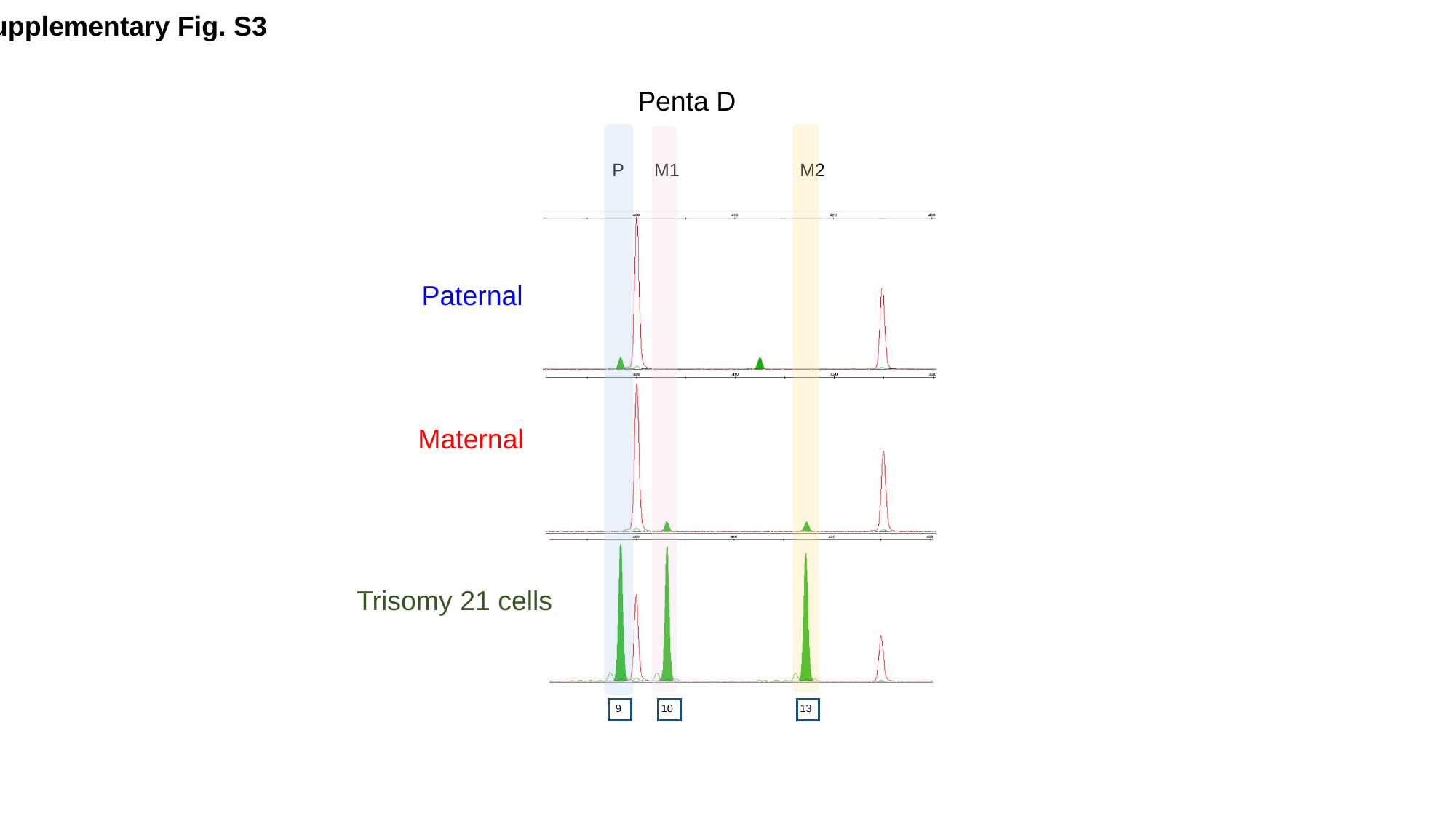

Supplementary Fig. S3
Penta D
P
M1
M2
Paternal
Maternal
Trisomy 21 cells
9
10
13

Supplement: Supplementary file 3 — Supplementary Fig.S3 [file 10038_2022_1049_MOESM3_ESM.pptx]

## Slide 1
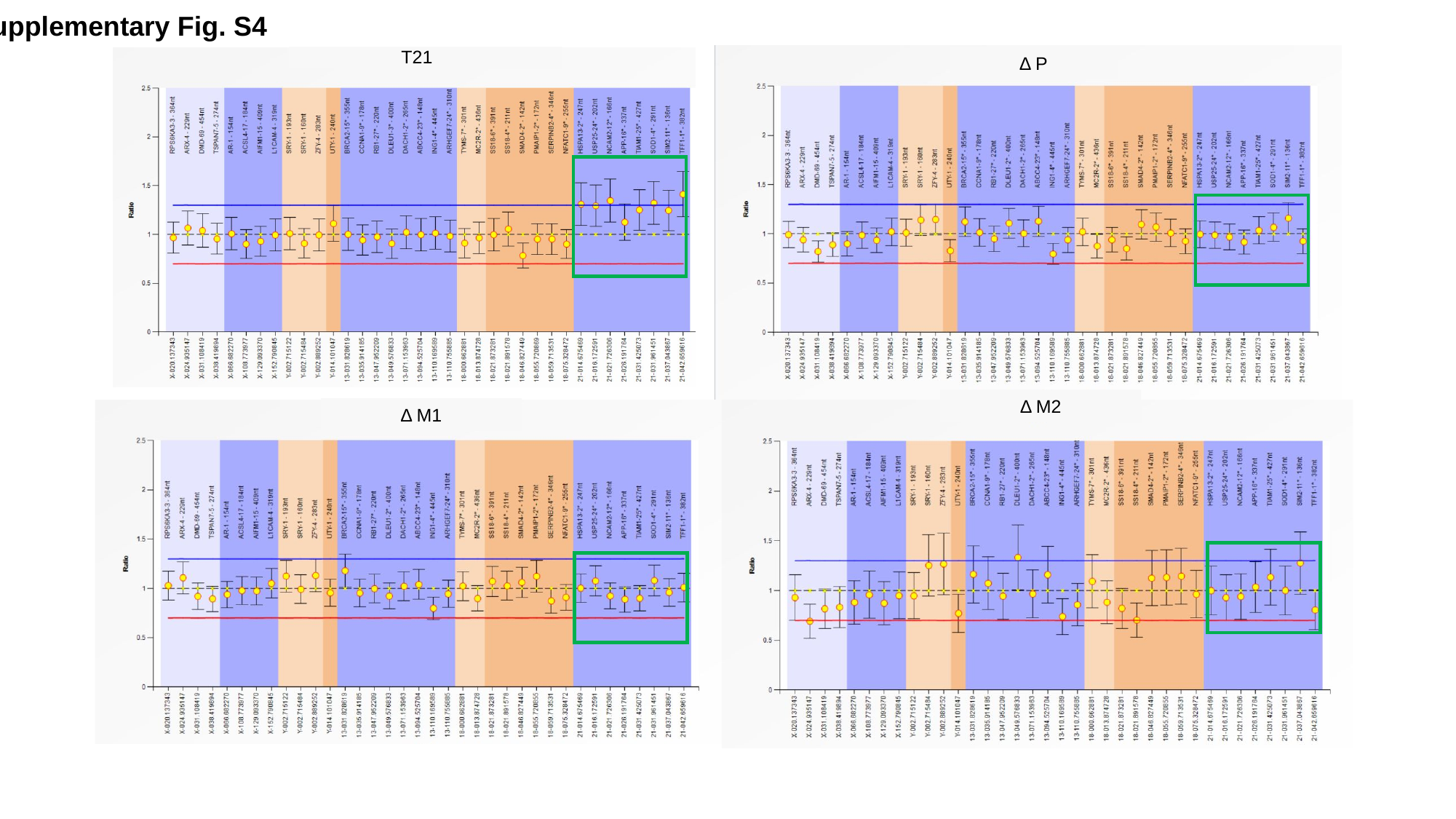

Supplementary Fig. S4
T21
Δ P
Δ M2
Δ M1

Supplement: Supplementary file 4 — Supplementary Fig.S4 [file 10038_2022_1049_MOESM4_ESM.pptx]
