# Supplementary material for: Experimental method for haplotype phasing across the entire length of chromosome 21 in trisomy 21 cells using a chromosome elimination technique
Source: J Hum Genet. 2022 May 31;67(10):565–72. doi: 10.1038/s10038-022-01049-6 (PMC9510051; doi:10.1038/s10038-022-01049-6)
Supplement: Supplementary file 5 — Supplementary Fig.S5 [file 10038_2022_1049_MOESM5_ESM.pptx]

## Slide 1
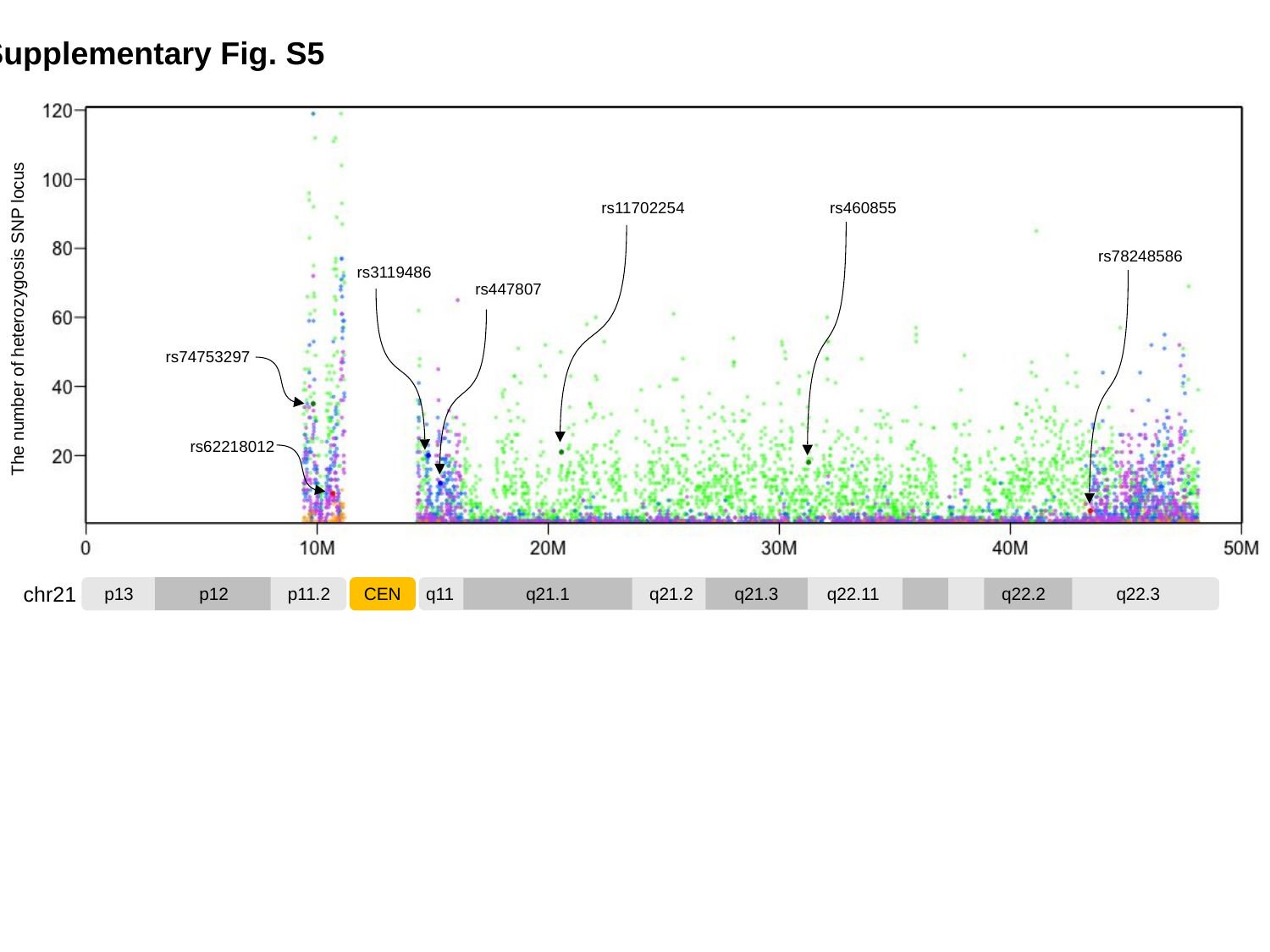

rs11702254
rs460855
The number of heterozygosis SNP locus
rs78248586
rs3119486
rs447807
rs74753297
rs62218012
chr21
p13
p12
p11.2
CEN
q11
q21.1
q21.2
q21.3
q22.11
q22.2
q22.3
Supplementary Fig. S5
