# Supplementary material for: Experimental method for haplotype phasing across the entire length of chromosome 21 in trisomy 21 cells using a chromosome elimination technique
Source: J Hum Genet. 2022 May 31;67(10):565–72. doi: 10.1038/s10038-022-01049-6 (PMC9510051; doi:10.1038/s10038-022-01049-6)
Supplement: Supplementary file 6 — Supplementary Table 1 [file 10038_2022_1049_MOESM6_ESM.docx]

**Supplementary Table 1** Primer sequences for STR analysis performed in this study.

| STR Marker | primer | Size of amplified product (bp) | Chromosome location |
| --- | --- | --- | --- |
| Penta-D  [1] | 5′-[JOE]-GAAGGTCGAAGCTGAAGTG-3′  5′-ATTAGAATTCTTTAATCTGGACACAAG-3′ | 370-454 | 21q22.3 |
| D21S11  [2] | 5′-ATATGTGAGTCAATTCCCCAAG-3′  5′-[JOE]-TGTATTAGTCAATGTTCTCCAGAGAC-3′ | 155-273 | 21q21.1 |
| D21S1411  [3] | 5′-ATAGGTAGATACATAAATATGATGA -3′  5′-[JOE]-TATTAATGTGTGTCCTTCCAGGC-3′ | 256–340 | 21q22.3 |

**Supplementary method**

Using Prime STAR MAX DNA polymerase (Takara Bio Inc., Shiga, Japan), PCR was performed according to the manufacturer’s protocol. The PCR program was set as follows: denaturation at 96°C for 1 min; 30 cycles at 94°C for 5 s, 60°C for 5 s, and 70°C for 10 s; and a final extension at 72°C for 1 min. Next, 16.5 µL Hi-Di Formamide and 0.5 µL Gene Scan Internal Lane Standard 600 (Promega, Madison, USA) were added to 1 µL of PCR amplified products. The mixture was loaded onto an ABI 3130 Genetic Analyzer (Applied Biosystems, Massachusetts, USA). The results were analyzed using Gene Mapper V4.0.

**Supplementary references**

1. <https://strbase.nist.gov/str_Penta_D.htm>

2. <https://strbase.nist.gov/str_D21S11.htm>

3. Paz N, Zabala A, Royo F, García-Orad Á, Zugaza JL, Parada LA. Combined fluorescent-chromogenic in situ hybridization for the identification and laser microdissection of interphase chromosomes. PLoS One. 2013;8:e60238.
